# A general formalism for the determination of the effective mass of the nanoscale structural inhomogeneities of the domain wall in uniaxial ferromagnets

**DOI:** 10.1186/s11671-015-0861-z

**Published:** 2015-04-02

**Authors:** Andriy Shevchenko, Maksym Barabash

**Affiliations:** G.V. Kurdymov Institute of Metal Physics, National Academy of Science of Ukraine, 36 Vernadskogo Pr., 03680 Kyiv−142, Ukraine; Technical Centre, National Academy of Science of Ukraine, 13 Pokrovskya Str., 04070 Kyiv, Ukraine

**Keywords:** 61.46. − w, 03.65. − w, 03.65.Db, Gyrotropic Thiele force, Uniaxial ferromagnet, The domain wall, The vertical Bloch line, The Bloch point, Gyrotropic bend, The effective mass

## Abstract

On the basis of the method of gyrotropic Thiele forces, we build a formalism that allows the determination of the effective mass of the nanoscales structural elements of the domain wall (DW): vertical Bloch line and Bloch point in uniaxial ferromagnets. As shown, the effective mass of these magnetic inhomogeneities depends on the value of the gyrotropic domain wall bend that is created by their movement.

**Electronic supplementary material:**

The online version of this article (doi:10.1186/s11671-015-0861-z) contains supplementary material, which is available to authorized users.

## Background

The study of structural localized inhomogeneities of the domain walls (DWs) of domain-containing magnetic materials is one of the topical problems in the solid state physics. Of special interest are the distributions of the magnetization vector $\overrightarrow{M}$ in the DWs of uniaxial ferromagnets: the vertical Bloch lines (BLs) and Bloch points (BPs) (see [1] and review [2]). The vertical BL and BP are stable nanoscale formations (approximately 10^2^ nm), which significantly affect the dynamics of the DW in external magnetic fields. Furthermore, BLs and BPs are considered as promising carriers of information in high performance memory devices with a superdense magnetic memory [3].

Many aspects related to the generation, stability, and dynamics of the vertical BL and BP have been studied. At the same time, some provisions that characterize the given inhomogeneities require more detailed consideration. One of these problems is to determine *m*_*L,BP*_ - the effective masses of the vertical BL and BP. The effective mass is an important dynamical characteristic caused by the quasiparticle approach to the investigation of vertical BL and BP movement. Accordingly, *m*_*L,BP*_ are determined from the ‘kinetic potentials’ constructed on the basis of systems of integrodifferential equation solutions of the BL and BP dynamics. This approach presents significant difficulties [4-7], and it would be laudable to build a simpler theoretical formalism for finding *m*_*L,BP*_ in the DW of different domain configurations. Another difficulty with the previous formalism is related to the dependence of the BL and BP parameters of the quantum effects, which take place for these magnetic structures in the subhelium temperature range, on their effective mass expressions [8,9]. This aspect is especially important for spintronics materials such as cylindrical nanowires and nanostripes in which is the presence of structures similar to BLs and BPs [10-13]. The construction of a general method, which allows the determination of the effective mass of nanoscale structural inhomogeneities of the DW in ferromagnets with strong uniaxial anisotropy, using the concept of a gyrotropic (gyroscopic) Thiele force acting on a moving magnetization distribution [14] is the aim of our work.

## Methods

### Problem solving and discussion

Let us consider an isolated domain wall containing vertical BL that separate DWs into regions with opposite orientations of magnetization. In the Cartesian coordinate system with an axis OZ directed along the anisotropy axis, and an axis OY - in the normal plane of the DW, the position of the vector $\overrightarrow{M}$ will be characterized by the polar and azimuthal angles *θ* and *φ*, respectively. The functional dependences of these angles corresponding to the static states of the DW and BL are well known and have the form [1]

$$ \theta (y)=\pm 2 arctg \exp \left(y/\Delta \right); $$

1 $$ \phi (x)=\pm 2 arctg \exp \left(x/\Lambda \right), $$

where **Δ** is the DW width, $\Lambda =\Delta \sqrt{Q}$ is the BL characteristic size, and *Q* is the material quality factor (the ratio of the magnetic anisotropy energy to the magnetostatic one), *Q* > > 1.

Assuming that the system considered here is stationary, the expression for the density of gyroscopic force ${\overrightarrow{\mathrm{f}}}_{\mathrm{g}}$ that acts on the system can be written as follows [14]

2 $$ {\overrightarrow{\mathrm{f}}}_{\mathrm{g}}=\frac{M_S}{\gamma}\left[\overrightarrow{\mathrm{g}}\times \overrightarrow{v}\right] $$

where *M*_*S*_ is the saturation magnetization, *γ* is the gyromagnetic ratio, $\overrightarrow{\mathrm{g}}=- \sin \theta \left[\overrightarrow{\nabla}\theta \times \overrightarrow{\nabla}\phi \right]$ is the gyrotropic vector, and $\overrightarrow{v}=\left({v}_x{\overrightarrow{e}}_x,\;{v}_y{\overrightarrow{e}}_y\right)$ is the velocity of the magnetic inhomogeneity.

It should be noted that the vector $\overrightarrow{\mathrm{g}}$ is a local measure of the heterogeneity of the moving magnetization distribution in two coordinates *x* and *y* (in given case) and characterizes the link between its parts - BL and DW. This allows us to consider the ${\overrightarrow{\mathrm{f}}}_{\mathrm{g},x,y}$ components as interaction forces. So, the ${\overrightarrow{\mathrm{f}}}_{\mathrm{g},x}$ component manifests as a force that acts from the moving domain wall on the vertical BL. In turn, the component of ${\overrightarrow{\mathrm{f}}}_{\mathrm{g},y}$ acting on the DW is caused by the dynamics of the BL. The result is a deformation of the DW, namely a gyrotropic bend that can be characterized in terms of the Slonczewski approach [1] by the coordinate of the normal displacement of its center - *q*(*ξ*), where *ξ* = *x* − *v*_*L*_*t*, and *v*_*L*_ is the BL velocity. In this case, external to the DW force ${\overrightarrow{\mathrm{f}}}_{\mathrm{g},y}$ produces the work with the average value $\overline{W}$ that is related to the kinetic energy of the BL as follows

3 $$ \overline{W}=\frac{1}{2}{\displaystyle \int {\mathrm{f}}_{\mathrm{g},y}} dxdy\;{q}_{\xi \to 0}=\frac{m_L{v}_L^2}{2} $$

Here *q*_*ξ* → 0_ is DW displacement in the BL center. It is this equation and will be the basis for finding the effective mass of the DW structural elements. We will consider its application on specific examples.

For DW stabilized by the external bias field gradient *H*_*g*_, the system of equations of DW dynamics has form [4]

4 $$ \begin{array}{l}\dot{\psi}\Delta {\omega}_M^{-1}={\Lambda}^2{\partial}^2q/\partial {x}^2\kern0.5em -\kern0.5em fq\\ {}\dot{q}{\Delta}^{-1}{\omega}_M^{-1}=-{\Lambda}^2{\partial}^2\psi /\partial {x}^2+ \sin \psi \cos \psi \end{array} $$

where *ψ*(*ξ*) is angle of the vector $\overrightarrow{M}$ in the DW center with axis OX, *ω*_*M*_ = 4*πγM*_*S*_, and *M*_*S*_ is the saturation magnetization, *f* = *H*_*g*_Δ/4*πM*_*S*_.

In the case *f* < <1 and *v*_*L*_ < <Λ*ω*_*M*_ and we obtain from (4)

5 $$ {q}_{\xi \to 0}=\pi \frac{\Delta {\omega}_M^{-1}{v}_L}{2\Lambda \sqrt{f}} $$

In the first approximation we assume that *ψ*(*ξ*) = *φ*(*ξ*) and using formulae (1) to (3), (5), we find the effective mass of the vertical BL

6 $$ {m}_L=\pi {\left(4\sqrt{f}{\gamma}^2{Q}^{1/2}\right)}^{-1} $$

Note that expression (6) is the same as the formula for *m*_*L*_ from article [4] lending credence to formalism presented here. In our case, we did not need the solution of the system Equation (4). We have found the asymptotic expression for the gyrotropic bend of the domain wall *q*_*ξ* → 0_, which simplifies the study of the problem.

## Results and discussion

The dependence of the effective mass on the DW gyrotropic bend value caused by the motion of the BL indicates an inertial character of *m*_*L*_. In this case, a decrease in the field of stabilization of the domain wall *H*_*g*_ should result in an increase of *q*_*ξ* → 0_ and, accordingly, *m*_*L*_. This agrees with formula (6): ${m}_L\sim 1/\sqrt{f}$. In turn, from the system of equations (4), it is easy to show that *m*_*L*_ ~ *f*^−1^ for values *f*> > 1. The dependence of the effective mass of the vertical BL on the gradient of the bias field stabilizing DW indicates the unstable natural motion of ‘hard’ DW (DW with complex interior structure), which is also reflected in the quadratic character of the DW spectrum oscillations [15]. Obviously, an external magnetic field is necessary for stabilizing the DW in the case of vertical BL motion.

It should be mentioned that taking together expression (2) and differentiating with respect to time of the second equation of system (4) yields, as expected, the Newton’s Second law for the DW

$$ {m}_{DW}\ddot{q}={\displaystyle \int {\mathrm{f}}_{\mathrm{g},y}}\kern0.15em  dy $$

where *m*_*DW*_ = (2*πΔγ*^2^)^− 1^ is the DW effective mass [1]. Note that the viscosity of the system is taken into account by introducing into the balance of forces a dissipative linear vector function [16], which, to simplify the consideration of the problem, we do not consider in our article. Its effect on the stationary motion of the BL and BP results in finite values of the mobility of the DW structural inhomogeneities, causing negligibly small additions to their effective masses [1,6,7]. However, in yttrium-iron garnets, where the viscosity is due to an exchange relaxation of the magnetization vector [17], the viscosity can considerably affect the BP dynamics [18]. Therefore, in these materials it is necessary to consider some given factor in the determining of the BP effective mass.

Let us now consider the vertical BL in the DW of the magnetic film. In this case, from article [6], it follows that the DW gyrotropic bend in the BL center has the form

7 $$ {q}_{\xi \to 0}={\dot{x}}_0{b}^{-1}{\omega}_M^{-1}\sqrt{\pi }{Q}^{-1/2}, $$

where *ẋ*_0_ is the BL velocity, *h* is film thickness,

$$ b={\left(f-{f}_c\right)}^{1/2}{\left(1+\frac{\Delta}{\pi h{k}_c^2}-\frac{\Delta}{\pi h}{\left(\frac{h}{\Lambda}\right)}^2{K}_0\left(\frac{k_ch}{\Lambda}\right)-\frac{\Delta}{\pi h}{\left(\frac{h}{\Lambda}\right)}^2\frac{K_1\left({k}_ch/\Lambda \right)}{k_ch/\Lambda}\right)}^{1/2}, $$

*f*_*c*_ and *k*_*c*_ are the respective critical values of the bias field gradient and wave vector characterizing DW bending instability [19], and *K*_0,1_(*x*) are the McDonald functions.

It is noteworthy that instead of the laborious process of finding the ‘kinetic potential’ of the vertical BL, it is possible to use Equations (3) and (7) to easily obtain the expression for *m*_*L,f*_ - the effective mass of the vertical BL in DW of magnetic film. The result (provide a reference to the final equation) coincides with the corresponding formula given in article [6]

$$ {m}_{L,f}=3\pi {\left(8b{\gamma}^2{Q}^{1/2}\right)}^{-1}. $$

Similarly, for the vertical BL in the domain wall of the magnetic bubble domain, in accordance with [7], defining the Fourier harmonics of the DW bend in the BL center, we can write

$$ {q}_{1,0}^{(1)}=-\frac{{\dot{\beta}}_L{a}^2h}{4{\omega}_M\left[{S}_0(a)-l{h}^{-1}\right]}\kern0.5em ,\kern0.5em {q}_{1,n}^{(1)}=-\frac{{\dot{\beta}}_L{a}^2h}{2{\omega}_M\left({n}^2-1\right)\left[l{h}^{-1}-S{}_n(a)\right]ch\left(\pi \Lambda n/2r\right)},\kern0.5em n\ge 2\kern0.15em ;\kern0.15em {q}_{2,n}^{(1)}=0, $$

where *β*_*L*_ is the angle coordinate of the BL center, *a* = 2*r*/*h*, *r* is the domain radius, *l* is the characteristic length, and *S*_*n*_(*a*) is the Thiele functions [20]. Then using Equation (3), we find that *m*_*L,BD*_ - the effective mass of the vertical BL in the DW of magnetic bubble - can be written as

$$ {m}_{L,BD}=\frac{a}{\gamma^2}\left[\frac{1}{4\left[{S}_0(a)-l{h}^{-1}\right]}+{\displaystyle \sum_{n=2}^{\infty}\;\frac{c{h}^{-2}\left(\pi \Lambda n/2r\right)}{2\left({n}^2-1\right)\kern0.5em \Big[l{h}^{-1}-{S}_n(a)}}\right] $$

The identity of the given formula to similar expression from article [7] again demonstrates the universality of our formalism in a variety of domain systems.

Let us consider the DW, where elements of the internal structure are the vertical BL and Bloch point, and separate the BL into two parts with an antiparallel orientation of vector $\overrightarrow{M}$. The characteristic area of BP is the domain wall region Δ < *R* < Λ, where $R=\sqrt{x^2+{z}^2}$. It is the area that mainly contributes to the effective mass of the Bloch point - *m*_*BP*_. In this region, there is a ‘vortex-like’ deformation of the BL magnetic structure, which is described by the system of equations [5]

8 $$ \begin{array}{l}\dot{\varphi}\Delta {\omega}_M^{-1}={\Lambda}^2{\partial}^2q/\partial {x}^2+{\Lambda}^2{\partial}^2q/\partial {z}^2\kern0.5em -\kern0.5em fq\\ {}-\dot{q}{\Delta}^{-1}{\omega}_M^{-1}={\Lambda}^2{\partial}^2\varphi /\partial {x}^2+{\Lambda}^2{\partial}^2\varphi /\partial {z}^2\end{array} $$

where *φ = arctgM*_*y*_/*M*_*x*_.

In the static state, the solution for the angle *φ*(*z*,*x*) can be defined from the second equation of system (8) and has the form

9 $$ tg\varphi =z/x $$

It should be noted that the direct application of formula (3) is limited by the vanishing of the magnetic moment vector in the BP center, making it necessary to use the microscopic Landau - Lifshitz Equation. However, since it is this area Δ < *R* < Λ that corresponds to core structural deformation of the Bloch line by BP, it is clear that Equation (3) can be used to estimate *m*_*BP*_. For this purpose, assuming *z* = *z* − *v*_*z*_*t* (*v*_*z*_ is BP velocity) from formulae (1) and (2), and taking into account expression (9), we find that the force causing the DW gyrotropic bend can be expressed as

10 $$ {\displaystyle \int dy{\displaystyle \underset{\Delta <R\le \Lambda}{\int }{\mathrm{f}}_{\mathrm{g},y}\kern0.15em  dxdz}=\frac{2{M}_S{v}_z}{\gamma }}{\displaystyle \underset{\Delta <R\le \Lambda}{\int}\frac{\partial \varphi }{\partial z}} dxdz\approx \frac{4{M}_S{v}_z\Lambda}{\gamma } $$

Further, from the first equation of system (9) for the coordinate of the DW normal displacement, we obtain $q\sim \pi \Delta {\omega}_M^{-1}{\Lambda}^{-1}{v}_Z$. Assuming that the bias field gradient is small *f* < <1, and using formulae (3) and (10), we find an estimation of the BP effective mass, which coincides with the corresponding result in the article [5]:

$$ {m}_{BP}\sim \Delta /{\gamma}^2 $$

An analysis of this expression shows that *m*_*BP*_ does not depend on the bias field gradient, in contrast to the dependence of the effective mass on the vertical BL (see expression (6)). This results from the local character of the DW area distortion by the Bloch point (~Λ^2^), the nature of which is determined by the surface tension of the domain wall (the terms ~ Λ^2^∂/∂*x*^2^ and ~ Λ^2^∂/∂*z*^2^ in the first equation of the system (8)). In turn, the deformation of the domain wall caused by the moving BL occurs along the entire line of the inhomogeneity, resulting in the displacement of the domain wall. Therefore, the vertical BL effective mass is determined by the bias field gradient stabilizing DW.

In the case of strong magnetic fields *f*> > 1, the singularity at the BP center can be eliminated by integrating over the BP volume. Indeed, from formula (2) and the first equation of the system (8), we define a gyroscopic force ${\mathrm{f}}_{\mathrm{g},y}=-\frac{M_S}{\gamma } \sin \theta \frac{\partial \theta }{\partial y}\frac{\partial \varphi }{\partial z}{v}_z$ and coordinate of the DW normal displacement $q=\Delta {\omega}_M^{-1}{f}^{-1}\frac{\partial \varphi }{\partial z}{v}_z$. Assuming the lower limit of the integral to be equal to Δ, we find and expression for work $\overline{W}=\frac{1}{2}{\displaystyle \int {\mathrm{f}}_{\mathrm{g},y}\kern0.15em  qdxdydz}$ and, using Equation (3), we arrive at an expression for the BP effective mass

$$ {m}_{BP}=\frac{\pi {M}_S}{H_g{\gamma}^2} \ln Q $$

The resulting expression differs only in terms of coefficients with the corresponding expression for *m*_*BP*_ in article [5]. The effective mass of the BP in this case is determined by the external field *H*_*g*_ and tends to zero with the DW gyrotropic bend at *f* → ∞.

These examples show that the formalism presented here, which incorporates our simple method for finding the effective masses of the vertical BL and BP, agrees well with all previously known results on this subject. Moreover, because of its generality, it can be extended to other ‘hard’ DW systems, such as dumbbell- and strip-shaped domains.

It should also be noted that expressions (2) and (3) are applicable to any moving magnetization distribution. Therefore, our formalism does not depend on the type of the ferromagnet or inhomogeneity. In this article, we have considered how local nanoscale inhomogeneities affect the interior structure of DWs in uniaxial ferromagnets classified as the vertical BL and BP.

## Conclusions

We have created a simplified formalism for finding of the effective mass of structural inhomogeneities of the DW - the vertical Bloch line and Bloch point in different domain systems of uniaxial ferromagnetic materials. It has been shown that the effective mass of the vertical BL and BP depends on the DW bend value and arises from their dynamics. At the same time, in the case of the BL, the gyrotropic bend of DW is determined by the external magnetic field stabilizing DW. In turn, the deformation of the DW due to the motion of the Bloch point is local in character and is caused both by the field stabilization of the DW and its surface tension.
